# Pertaining to Pyorrhea

**Published:** 1909-09-15

**Authors:** J. D. Patterson


### PERTAINING TO PYORRHEA.

BY J. D. PATTERSON, D.D.S.

Read before the Kansas City Dental Society. Answers to List of Questions Sent Out by The Louisiana State Dental Association.

*First.—Do you consider pyorrhea alveolaris incurable?*

This first question must be answered somewhat evasively. In a case of this nature when there has been destruction of function with accompanying loss of soft and hard tissue following the processes of inflammation, which is denoted by gum recession and solution of bony process about and beyond the cervix of the tooth, there will be no reproduction of this tissue in semblance of its original anatomical perfection. In fact, all methods of sponge grafting or by surgical or the other similar measures endeavoring to coax the tissue to assume its shape have proven failures. Indeed, with the best methods of treatment there is usually in advanced cases, a still further shrinking of gum tissue in the healing process—so, if the question demands for answer that a cure shall restore tissue lost we must answer that this cannot be. However, from the standpoint of the surgeon in conditions of this character, such as catarrh, if he succeeds in removing disease causes and all inflammation, exudations, pain and discomfort, he has, he says, arrived at a cure, although the tissue on account of long loss of function and inflammatory procedures may never closely resemble that loss, or functionate physiologically. So, in the disease of pyorrhea alveolaris, we may with perfect confidence say that a cure has been effected when the inflamed and painful tissues about the diseased tooth take on a healthy hue, are free from inflammation, exudation and pus, the pain has ceased, the germs cling close to the cervix and comfort has come to take the place of discomfort. Therefore to the second question, viz.:

*Second.—Do you know of any cases that have been cured?*

We can answer most assuredly that very many cases are cured.

*Third.—In what stages were these cases when first observed?*

A cure can be brought about where there is a considerable amount, say one-third, of the normal attachment remaining; but cures, whether in incipient or advanced stages, must ever depend upon whether the patient can or will permit thoroughness in the surgical operation and maintain subsequently the most scrupulous hygenic care.

*Fourth.—Have you ever recognized the disease before serumal calculus appeared?*

Believing that serumal or sanguinary deposits are subsequent to the initial irritation, we would say that the first stages may often be observed as a simple gingivitis form, a variety of causes with no deposit as yet from the serum. The point at which calcic matter from the exudates may first appear is not easily determined, however.

*Fifth.—What were the symptoms?*

Simple gingivitis, from fermentation, putrefaction, salivary deposits, mouth breathing or other irritation to the gingival margin at the cervix of the tooth.

*Sixth.—Do you think salivary calculus has any effect on the disease?*

Salivary calculus from the irritation to the gum by its mechanical touch, and by the nests of fermentation and putrefaction it fosters, causes no doubt about ninety per cent. of pyorrhea cases.

*Seventh.—Were the patients under your observation of robust or delicate physique?*

This question needs no answer as one with a "delicate physique" may be in as perfect physical condition as the robust. Instead of the word "physique," however, I apprehend the committee meant health, or condition and to that I would say that the great majority of pyorrhea patients are without systemic predisposition.

*Eighth.—Have you ever seen the disease in youth, and at what age?*

Very few under twenty years of age and in these mouths much filth on account of decayed teeth preventing mastication.

*Ninth.—Do you know of any properly devitalized teeth being lost by this disease?*

Yes.

*Tenth.—Did the devitalization and root canal sterilization and filling precede or follow pyorrhea development?*

Both.

*Eleventh.—At what time of life do you find treatment most efficacious?*

At the time of life when the patient becomes thoroughly imbued with the fact that only the most strenuous endeavors will prevent the loss of the dental organs.

*Twelfth.—Do you find pyorrhea more in males or in females?*

About the same.

*Thirteenth.—Do you find it more in the upper or lower jaw?*

About the same.

*Fourteenth.—Does the disease attack all teeth alike?*

No.

*Fifteenth.—Were patients ever afflicted with syphilis, tuberculosis, uricæmia, chronic indigestion or chronic constipation?*

Perhaps about three per cent. with syphilis, six per cent. with tuberculosis, five per cent. with uricæmia, and the Lord knows how many with chronic indigestion or constipation.

*Sixteenth.—Have you ever seen a case where malocclusion was a cause?*

Yes; because it renders cleansing by mastication or the brush well nigh impossible. I have also seen many cases where the cause of malocclusion was pyorrhea.

*Seventeenth.—Do you think auto-intoxication a cause?*

Systemic conditions alone without local irritation are rarely if ever a cause of pyorrhea. On the other hand, copious amounts of pus from pyorrhea taken into the alimentary canal, by auto-intoxication, may cause uricæmia.

*Nineteenth.—Is it a disease of the gums?*

Sure, primarily.

*Twentieth.—Is it a disease of the alveolar process?*

The alveolar process becomes involved.

*Twenty-first.—Is it a disease of the peridental membrane?*

With the progress of the disease to the peridental membrane, all tooth investment becomes involved.

Concluding brief answers to the questions sent abroad by the Louisiana State Dental Society, I am asked by the tenor of the notices sent out by the program committee for this meeting to deal with the practical treatment or cure of this disease, while to another has been given consideration of prevention, or prophylaxis.

I will then confine myself in conclusion to the methods which have been found in effecting cures in the baffling and prevalent malady. It may be said that corrective measures bringing cures cannot be divorced from a well-grounded knowledge of the disease causes. *True, every time,* and while not dealing at this time with what I deem the various etiologic factors, I will, as directed, endeavor to briefly outline what the practical demands are upon us who desire in the most intelligent way to give to those suffering from this disease our best skill.

I think it may be adjudged by all in this audience that the destruction of the surrounding membranes of the dental organs in the disease we are placed to call pyorrhoea alveolaris, results largely from local unsanitary conditions. I think that those who are insistent upon systemic causation, whether of the uric acid diathesis--gout, rheumatism, urecæmia, faulty metabolism, syphilis, tuberculosis, arthritis, etc., and who insist, or recommend, that in treatment, systemic remedies and vaccines are essential, will most generally concede that thousands of cases have been stopped from further ravages, and every non-hopeless case relieved, by a strict attention to the removal of all local irritation.

Not for a moment do we undervalue the systemic treatment which may aid in the recovery, and the permanency of such recovery, by reenforcing the economy with the ability to neutralize, or withstand, or destroy invading infection and irritation, but I think at the same time we must concede that tissue anemic from long deprivation of proper nutrition, until below the normal index of fighting capacity, is not rapidly brought to a better ability by therapeutics or vaccines, but that such ability must come through a reorganization more basic, more subtle, more prolonged, and as the result of physical exercise, good digestion, good nutrition, sunshine, sleep and good nervous equilibrium.

Therefore: In the meantime let us *at once* avail ourselves of local methods which *we know* bring almost instant and lasting response, viz.: the positive removal of local irritation and infection and the establishment of perfect sanitation. This it seems to me is sense and logic. *It certainly has secured results.*

That I may not weary you, or repeat what has been so often expressed or written, I will only add a method of procedure in a given case as follows:

1. A glance will tell the experienced whether treatment is demanded or not.

2. Obtain any history of each individual case as to family predisposition, previous treatment, etc.

3. The patient is made to vigorously rinse the mouth with a solution of permangate of potassium.

4. The operator flushes from the diseased territory all inflammatory products, nests of fermentation and putrefaction with hot water charged with an antiseptic.

5. Explore diseased places and pockets with a delicate probe to determine the extent of the progress of distruction.

6. Remove hopeless teeth, *i. e.*, those from which all normal attachment is gone and only the ligamentous remains.

7. Ascertain in the teeth that remain if the pulps are in a pathological condition. If so, remove them.

8. If teeth are very loose, and yet give promise of repair, place upon them splints to rigidly hold them in their proper places.

9. Obtund the tissue. Do not inject.

10. Remove all deposits. Stop when you do this; do not go deeper than the surface upon which the deposits are attached. *Leave the surface smooth.—Hartzell's Investigations.*

11. Remove all debris from the operation with strong force syringe, hot water and antiseptics.

Polish all surfaces operated upon with the various polish ing devices.

13. Bathe all sensitive surfaces of teeth with a five per cent solution of silver nitrate. Posterior sensitive surfaces can be bathed with a ten per cent. solution or in extreme cases with the saturated solution.

After a few minutes use as a soothing and absorbent and stimulant application: 1 part carbolic acid, 2 parts tincture of aconite and glycerine, and three parts tincture iodine.

15. Give to your patient the pamphlet upon "The care of the Mouth and Teeth," published by the National Dental Association, and practically demonstrate the use of the brush upon a dummy, also methods of massage.—*Western Journal.*

